# Judging a salmon by its spots: environmental variation is the primary determinant of spot patterns in *Salmo salar*

**DOI:** 10.1186/s12898-018-0170-3

**Published:** 2018-04-12

**Authors:** Katarina M. Jørgensen, Monica F. Solberg, Francois Besnier, Anders Thorsen, Per Gunnar Fjelldal, Øystein Skaala, Ketil Malde, Kevin A. Glover

**Affiliations:** 1 Institute of Marine Research, Postboks 1870, Nordnes, 5817 Bergen, Norway; 2 Sea Lice Research Centre, Dept. of Biology, University of Bergen, 5020 Bergen, Norway; 3 Institute of Marine Research, Matre Research Station, 5984 Matre, Norway

**Keywords:** Atlantic salmon, Introgression, Environment, Genetics, Aquaculture, Interaction

## Abstract

**Background:**

In fish, morphological colour changes occur from variations in pigment concentrations and in the morphology, density, and distribution of chromatophores in the skin. However, the underlying mechanisms remain unresolved in most species. Here, we describe the first investigation into the genetic and environmental basis of spot pattern development in one of the world’s most studied fishes, the Atlantic salmon. We reared 920 salmon from 64 families of domesticated, F1-hybrid and wild origin in two contrasting environments (Hatchery; tanks for the freshwater stage and sea cages for the marine stage, and River; a natural river for the freshwater stage and tanks for the marine stage). Fish were measured, photographed and spot patterns evaluated.

**Results:**

In the Hatchery experiment, significant but modest differences in spot density were observed among domesticated, F1-hybrid (1.4-fold spottier than domesticated) and wild salmon (1.7-fold spottier than domesticated). A heritability of 6% was calculated for spot density, and a significant QTL on linkage group SSA014 was detected. In the River experiment, significant but modest differences in spot density were also observed among domesticated, F1-hybrid (1.2-fold spottier than domesticated) and wild salmon (1.8-fold spottier than domesticated). Domesticated salmon were sevenfold spottier in the Hatchery vs. River experiment. While different wild populations were used for the two experiments, on average, these were 6.2-fold spottier in the Hatchery vs. River experiment. Fish in the Hatchery experiment displayed scattered to random spot patterns while fish in the River experiment displayed clustered spot patterns.

**Conclusions:**

These data demonstrate that while genetics plays an underlying role, environmental variation represents the primary determinant of spot pattern development in Atlantic salmon.

**Electronic supplementary material:**

The online version of this article (10.1186/s12898-018-0170-3) contains supplementary material, which is available to authorized users.

## Background

Animal coats display a fascinating diversity of patterns across different species: the stripes and spots of cats, giraffes and zebras, the symmetry and colour of butterfly wings, and exotic swirl patterns on shellfish and fish [1]. Morphological colour changes in fish occur from variations in pigment concentrations and in the morphology, density, and distribution of chromatophores in three dimensions in the integument of the skin. Despite the wide variety of colours and patterns observed, the structural organization of pigments in teleost fish display similar features, suggesting a common mechanism of development and colour revelation [2].

All vertebrate chromatophores originate from the neural crest in the embryo, formed shortly after gastrulation [3]. The birth, migration, and differentiation of chromatophores is tightly regulated by signalling pathways and transcription factors [2, 3]. Although at least 100 genes involved in patterning in vertebrates have been identified, little is known about the molecular or cellular processes that generate complex patterns, or how alterations in those processes might produce different phenotypes [3]. Recent experimental studies [4–6], notably also in zebrafish (*Danio rerio*), point to the mechanism being Turing pattern formation, acting through differences in diffusion or cell–cell communication and reaction across the fish skin surface [7–9].

The pigment patterns of wild animals, including fish, are of importance to their fitness and serve a variety of functions in predator avoidance, prey capture and social communication [2, 3]. Melanin-based colouration may co-vary with traits such as dominance, stronger immune responses, and greater stress resistance [10]. Present evidence also suggests that fitter, dominant fish are spottier [11]. Pigment phenotype, or “spottiness” as it is commonly known, is often determined by polymorphisms in the melanocortin-1 receptor (MC1R) in vertebrates, and this receptor family is also involved in regulating immunity and stress responses [3, 10]. The specific role of melanophores is to protect the skin from UV damage [10]. In anadromous fish, such as Atlantic salmon (*Salmo salar* L.), plasticity of patterning is probably the consequence of ontogenic habitat shifts [12]. Patterning is a sequential process where the coat markings (known as ultimate patterns) repeatedly change as a result of a series of transitions between life stages such as parr/smolt and immature/nuptial [2]. At the present, only general information is available about skin colours and patterns in salmonids, and their genetic basis and environmental triggers are largely unknown [13]. One exception is the fine-spotted pigmentation pattern observed in some populations of brown trout (*Salmo trutta* L.) which has been shown to be inherited in a simple Mendelian pattern [14, 15]. Quantitative trait loci (QTL) analysis can assist in understanding the relationship between inheritance and phenotype and is an early step in identifying key genes [16]. Life-stage skin colouration can also be modulated in response to environmental factors, allowing fine-tuning of the ultimate patterns. Environmental factors can be primary when they have a direct effect on chromatophores, or they can be secondary, allowing further adjustment of colour during a life stage [2].

Atlantic salmon are characterized by a wide variety of genetic-based life-history, phenotypic and morphological diversity within and among populations [17]. This species has also been subject to domestication selection since the early 1970s [18], and as a result, domesticated salmon display a wide variety of genetic differences to wild salmon [19]. Notably, growth rate under controlled farming conditions is now several-fold higher than for wild salmon [20, 21]. In addition, variation in spot patterns between domesticated and wild salmon have been reported, with domesticated salmon displaying more spots [22, 23]. However, whether the difference in spot patterns between domesticated and wild salmon results from environmental or genetic variation remains to be determined. Salmon are easy to rear, and common-garden experiments can be conducted in a variety of environments ranging from farms [21, 24] to natural river systems [25, 26]. Such studies provide the ability to disentangle the relative roles of genetics, environment, and developmental triggers for phenotypic traits, such as spot patterns. In the present study, we aimed to determine whether the reported difference in spot patterns between domesticated and wild salmon is caused by genetic or environmental variation. In order to address this, we reared pedigree-controlled domestic, F1 wild-domesticated hybrid and wild salmon in both a natural river environment, as well as a hatchery, and quantified their spot patterns using a mixture of manual and automatic spot counting methods.

## Methods

### Overall study design

**Fig. 1:**
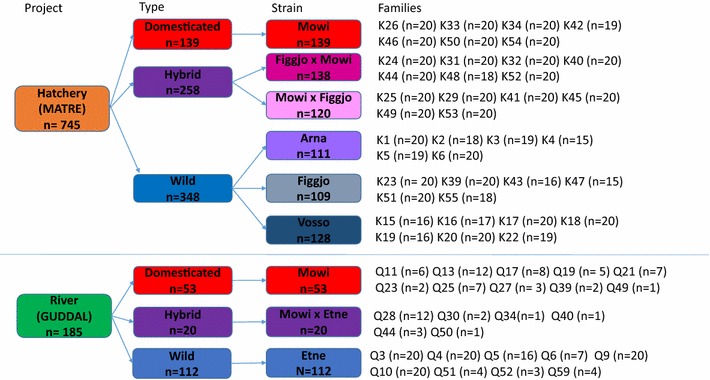
Experimental design. Overall design of both experiments. The colour scheme in this diagram explains the colours used in all other graphs in this study

This study is based upon an analysis of data from two experiments (Hatchery and River). In the Hatchery experiment, 2249 fish originating from 39 mostly full-sibling families of a domesticated, two F1 hybrid and three wild populations were reared under standard farming conditions using land-based tanks and sea-cages for the freshwater and marine stages of development respectively, and phenotyped as immature fish in sea cages at age 2+. In the River experiment, 344 fish originating from 25 mostly full-sibling families that were originally planted in the river Guddal as eyed eggs, hatched naturally and smoltified in the river at age 2–4, captured on a downstream trap upon seaward migration, and thereafter reared in tanks until phenotyping at age 4 and 6+. The numbers of fish and families involved, and the experimental pedigree design is presented (Fig. 1; Additional file 1). It is worth noting that the same domesticated strain was used in both experiments, although the wild populations varied. Phenotyping included taking growth measurements and photographing all fish. Linkage mapping analysis was performed on the fish reared in the Hatchery experiment in order to try to identify potential quantitative trait loci (QTL) associated with spot pattern variation.

### Production of the fish for the Hatchery experiment

**Fig. 2:**
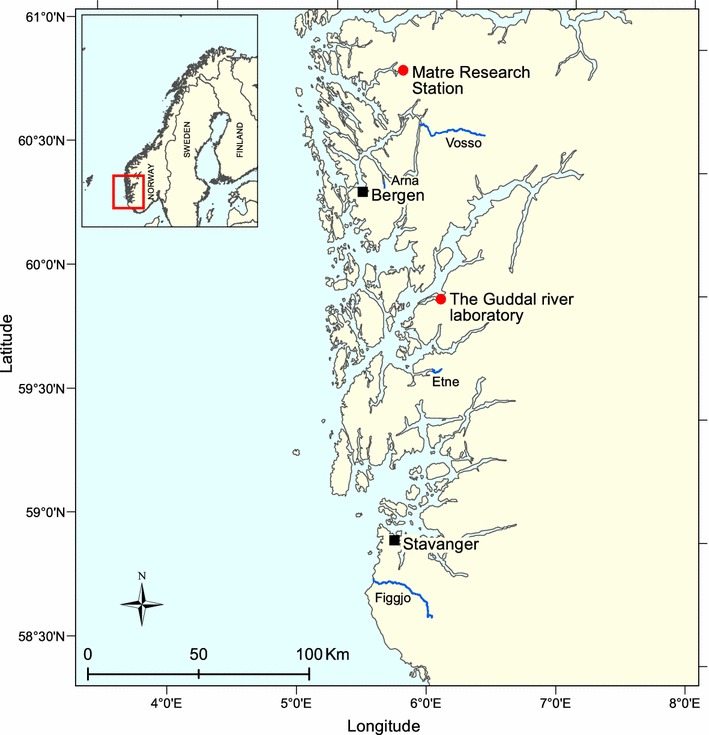
Map of research stations and rivers. Red dots mark the sites for the Hatchery (Matre) and the River (Guddal) experiments. Blue lines mark the rivers where the wild salmon in both experiments (Hatchery: Arna, Figgjo, Vosso, River: Etne) were obtained

Domesticated, wild and F1 hybrid salmon were produced under standard farming conditions at the Matre research station in Norway (Fig. 2). Wild parental salmon, captured in their respective rivers, were used to produce the experimental crosses in autumn 2011. These fish originated from the rivers Figgjo (58°81′N, 5°55′E), Arna (60°24′N, 5°29′E) and Vosso (60°64′N, 5°95′E) (Fig. 2). In addition, gametes of domesticated salmon, representing the ~ 11th generation of the Mowi strain owned by Marine Harvest, were collected from their main breeding station. We created a total of 39 families of domesticated and wild, in addition to reciprocal Figgjo × Mowi hybrids in November 2011 (Fig. 1; Additional file 1). More extensive details of these crosses can be found elsewhere [27]. All families (6–7 families/strain) were communally-reared in two replicates (N = 50 individuals/family/replicate) under identical fresh water tank conditions from the eyed-egg stage in February 2012 and onwards. In March 2013, these fish were adipose fin clipped, individually PIT tagged, and thereafter transferred to open net pens for further seawater rearing, 1200 smolts/replicate (initial n = 2400). Early maturation in the sea cages, i.e., jacking, was registered in January 2014. Finally, the fish were phenotyped in April 2014 at the age of 2+. During production, eggs were kept in the dark until they were ready for start-feeding. The survival rate of the eggs until hatching was ~ 99% [27]. Fish were reared on commercial pellets from Skretting. The fish experienced a 24-h light regime for the first 6 months, then a natural day-length for Bergen (60°20′N, 5°20′E) until transfer to sea cages. Osram L18W/865 Lumilux Cool Daylight, 1300 lm (UV free light) were used as the light source in fish tanks. Tank colour was green followed by grey. Light was natural in the 5 m deep cages.

### Production of the fish for the River experiment

Eyed eggs of domesticated, wild and F1 hybrid salmon families were planted into the river Guddal (61°14′N, 5°37′E) in 2008 and 2010 (Fig. 2). The planted families were established from broodfish captured in the river Etne (59°40′N, 5°56′E), as well as the domesticated Mowi strain, and their F1 hybrids. Eyed-eggs from 17 families (2–8 families per cross, family range Q1-30) and 29 families (9–10 families per cross, family range Q31-60) were planted in the river in April 2008 and 2010, respectively (Fig. 2; Additional file 1). This natural river system has previously been used for common-garden experiments involving planting out genetically diverse fish that are subsequently identified by DNA parentage testing [25, 28]. The planted eggs hatched naturally, and juveniles competed for resources until they smoltified at age 2+ and 4+. The river Guddal is a summer-cold river as it receives run-off from the Folgefonna glacier, limiting growth rates. Average survival from eyed egg to the smolt stage in this river is typically 1–2% based upon previous experiments [25]. Upon smoltification in May 2012, we caught a random sample (initial n = 379 consisting of 4 year smolts representing 16 families from cohort 2008 and 2 year smolts representing 9 families from cohort 2010) of the surviving fish planted into the river in a downstream trap, and thereafter transported them to the Matre fish farm where they were placed into 1.5 m^2^ rearing tanks with saltwater. At Matre, these fish were weaned onto Skretting pellets. These fish were reared in saltwater tanks from May 2012 onwards, fin clipped and PIT tagged in March 2013 and phenotyped for this study in April 2014 at the age of 4+ and 6+. In the river, salmon were exposed to natural light and had access to natural hiding places with shade. After being transferred to Matre, the salmon were held in tanks experiencing the natural day-length for Bergen. Tank colour was white for the first year (May 2012 to February 2013), and green during the second year. Osram L18W/865 Lumilux Cool Daylight, 1300 lm (UV free light) were used as the light source in fish tanks.

### Phenotyping fish from both experiments

In April 2014, salmon from the Hatchery (n = 2249) and River (n = 344) experiments were sampled. Fish were first anaesthetized with metacain (Finquel^®^ Vet, ScanVacc, Årnes, Norway), thereafter identified by their PIT tag, measured, and then photographed using a digital Canon EOS 650D camera. DNA parentage testing with microsatellites was used to identify all fish to their families and thus strains/populations of origin. Extensive details with respect to the exact genotyping procedure are available elsewhere [29].

From the Hatchery experiment, up to 20 individuals per family were selected for the image analysis (Fig. 1) using random selection in R version 3.2.2 [30]. In the River experiment, we used the same criteria for selection of individuals as above, however, many families were represented with a low number of individuals (reflecting differences in family survival in the river as has been observed previously [25]). Among the fish randomly chosen for image analysis from the Hatchery experiment, early maturing fish (n = 33) were excluded from the analysis. Early maturation was not registered for fish from the River experiment. Thus, the total number of individuals used for image analysis were 745 and 185 for the fish originating from the Hatchery and River experiments respectively. All raw data are presented (Additional file 2).

### Quantification of spots

**Fig. 3:**
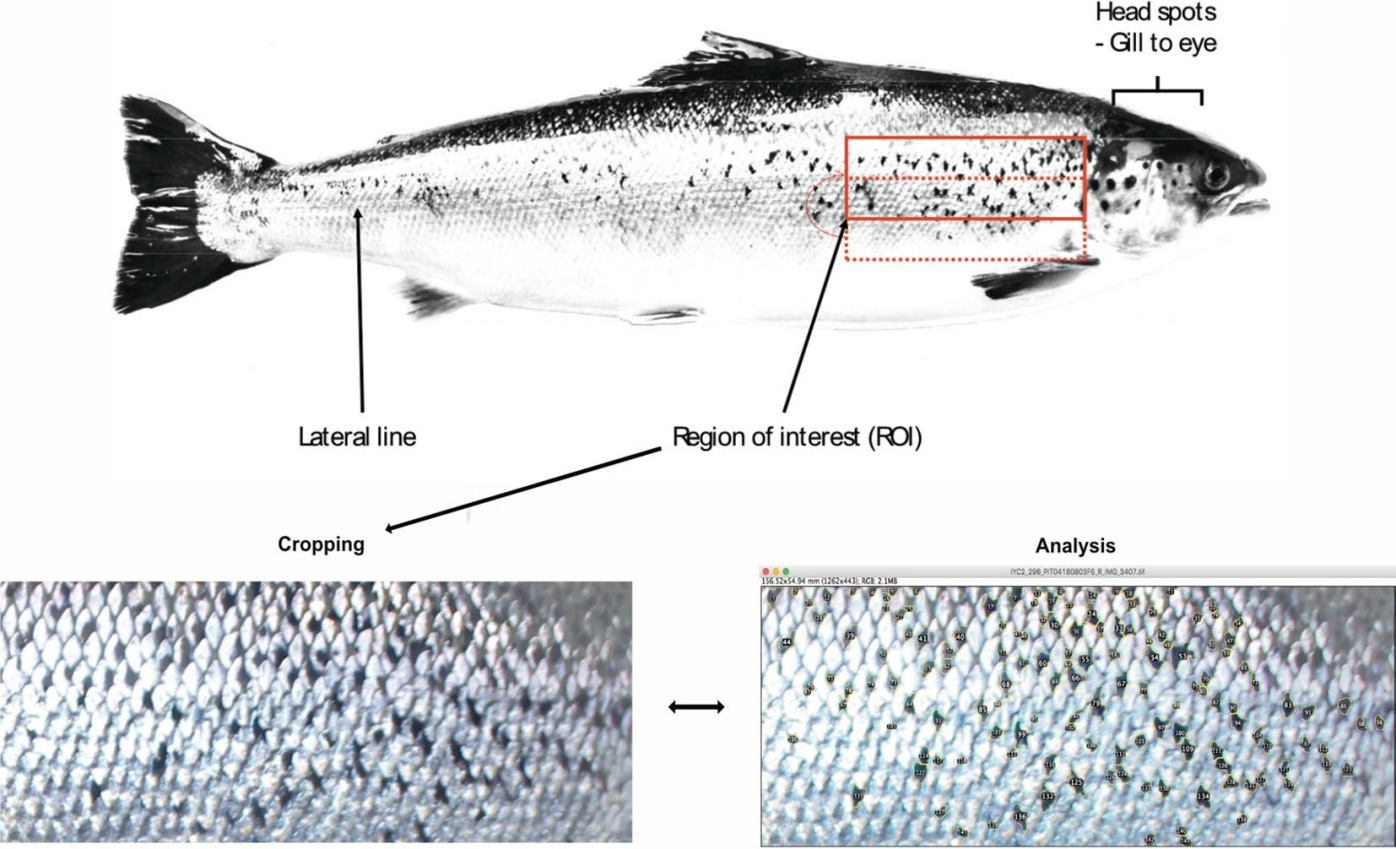
Spot sampling. (Top) A region of interest (ROI) around the lateral line is selected in the image analysis tool for spot counting. Counts in the head region are manual. (Bottom) The ROI is cropped and saved (left) and then processed by the macro for counting (right)

Spot patterns were quantified using both manual and automatic methods as described below. For the automatic counts, a standardized rectangular region of interest (ROI) was established. The ROI was placed just above the pectoral fin, by the gills, and then extended up to the lateral line (see Fig. 3, top). The length of the ROI was then extended to the front of the dorsal fin before the ROI was moved upwards until it is centred about the lateral line. Macros to count the numbers of spots within the ROI were created in the image analysis program ImageJ version 1.51 g [31] (Fig. 3, bottom, Additional file 3). The information extracted from the ROI included: spot number, Area, X-coordinate, Y-coordinate. The clustering coefficient (i.e., a measure of the spot aggregation/dispersion) was calculated using the Average Nearest Neighbour distance (ANN) [32].

In addition to automatic spot counts, human judgement was used to assess the overall spottiness of the fish. This counting method mirrored the results of the automatic method, and therefore, these data are not presented. The number of spots on the fishes’ head between the gills and the crease behind the gills, and from the crease to the eye of the fish were counted manually (Fig. 3, top). The entire head of the fish was not always represented in the photograph, and consequently, there were missing values: Hatchery (n = 4), River (n = 54).

### QTL analysis

All salmon from the Hatchery experiment were subject to linkage mapping using an identical approach to that described in [33]. In short, this included analysis of genome-wide distributed SNPs and associating their genetic variation to measurements of spottiness. An initial set of 118 SNP markers (Additional file 4) were selected as good candidates to cover the salmon genome at regular 20–30 cM intervals based on the salmon linkage map [34], and for having provided the best information content in a similar experimental design [33]. After genotyping was performed on a MassARRAY Analyzer 4 (Agena Bioscience™), a few of the SNPs did not amplify. This left the final number of SNPs at 109. The raw data was processed in Typer 4.0.20. The phenotypes used for the QTL analysis are found in Additional file 5, and the genotypes in Additional file 6. Genes in the detected linkage group can be found in Additional file 7. Genes identified by KEGG as participants in melanosome biogenesis are found in Additional file 8.

To perform QTL mapping, we first reconstructed the haplotypes of both parents and offspring based on pedigree and genotype data. The next step consists in estimating the Identity By Descent (IBD) coefficient between each pair of individuals at each locus along the genome. IBD coefficients were obtained from a recursive approach [35] implemented to account for haplotype information as input.

The QTL scan was then performed by fitting a Hierarchical Generalized Linear Model (HGLM) at each genomic location as:

1 $$y = X\beta + Ga + Zq + e$$

where *y* is the phenotype vector, *X* the design matrix for fixed effects, β the vector fixed effects, *G* the kinship matrix, *a* the vector of random polygenic effects, *Z* the design matrix for the QTL effect, *q* the vector of random QTL effect, and *e* the random residuals. Note that GG′ is equivalent to the square kinship matrix, and covariance structure for the random polygenic effects, and ZZ′ is equivalent to the square IBD matrix and covariance structure of the random QTL effects.

At each tested genomic position, the likelihood of model 1 is compared to the likelihood of the model without QTL effect:

2 $$y = X\beta + Ga + e$$

In both models, we consider the adjusted profile log-likelihood profiled over random effects as provided by HGLM [30, 36]. The likelihood ratio between model 1 and model 2 is then considered as the indicator for QTL i.e., correlation between genotype and phenotype variance.

To account for multiple testing along the genome, the genome wide significance threshold for likelihood ratio was obtained through a permutation test as in Churchill and Doerge [37].

### Statistical analysis

All data processing was carried out in R version 3.2.2 [30]. Correlation between response and explanatory variables was estimated using anova and linear models (not indicated in main text when used). To estimate the variance components of spot density, a mixed model was fitted using the lmer() function from the lme4 package [38]. A Kruskal–Wallis rank sum test was employed when testing the effect of strain on categorical data (spottiness, spots below lateral). Mann–Whitney (for categorical data) and T tests (for numerical data) were used for direct comparisons between the Hatchery and River experiments, and between the domesticated populations (Mowi) in the Hatchery and River experiments. Fish with no spots were excluded [missing in the Hatchery experiment n = 6 (4 Mowi, 2 Arna), River experiment n = 3 (all Mowi)] from analysis of automatic count variables (ROI size—and therefore all spot densities corrected for this), since these depend on the presence of spots. In the analysis of ANN values, 10 spots were chosen as an arbitrary threshold for exclusion, since whether the spots are near or far apart is random at low spot numbers [fish excluded in the Hatchery experiment n = 37 (9 Mowi, 2 F × M, 4 M × F, 11 Arna, 6 Figgjo, 5 Vosso)], River experiment [n = 24 (15 Mowi, 4 M × E, 5 Etne)]. In the analysis of influence of family on spot density, families with only one member were removed in the River experiment [n = 4, Q49 (Mowi), Q34, Q40, Q50 (M × E)]. The box plots used for data display are R default.

## Results

### Initial observations

**Fig. 4:**
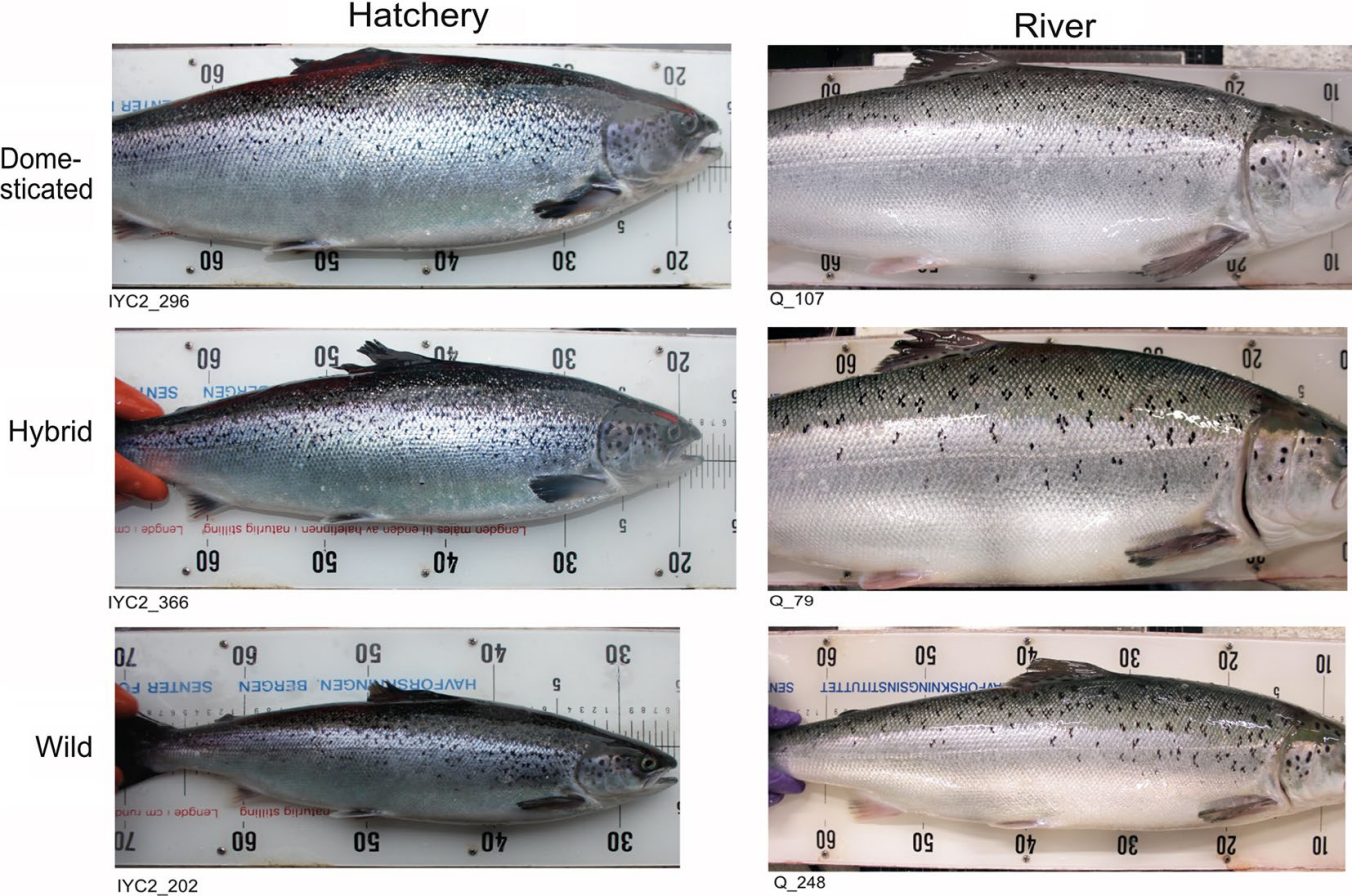
Spot patterns of Atlantic salmon primarily vary with environment, and not with genetic background. The Hatchery experiment (left) and the River experiment (right) and strain (top to bottom)

In both experiments, differences in spot patterns were not noticeable by eye among domesticated, hybrid and wild strains (Fig. 4). In contrast, and independently of genetic origin, clearly-visible differences in spot patterns between fish reared in the Hatchery and River experiments were observed (Fig. 4). In the Hatchery experiment, spots as represented by melanocytes (black cells) appeared more numerous and scattered on the fish (see also Fig. 3), while in the River experiment, spots appeared fewer and more clustered together to form geometric shapes such as diamonds or polygons, creating the appearance of larger spots with more space between them.

### Spot counts, fish weight and spot density

Spottiness varied significantly with strain in both experiments. Domesticated (Mowi) and hybrid fish [Mowi × Figgjo (M × F) and Figgjo × Mowi (F × M)] displayed more spots than wild fish (Arna, Figgjo, Vosso) in the Hatchery experiment (Table 1, strain significantly explains number of spots, R^2^ = 0.09, p = 6.7e−16). In contrast, fish of wild parentage (Etne) displayed more spots than the fish of domesticated origin (Mowi) in the River experiment (Table 1, strain significantly explained number of spots, R^2^ = 0.17, p = 2.7e−18). Fish from the Hatchery experiment displayed an average of 71.9 (± 44.1, range 0–237) spots, while fish from the River experiment displayed an average of 29.8 (± 18.1, range 0–107) spots (sign. difference between environments, t test t = 20, df = 718.6, p < 2.2e−16, all spot counts are automatic counts within a region of interest). The domesticated Mowi strain reared in the Hatchery experiment had significantly more spots than Mowi fish reared in the River experiment (Table 1, t = 11.46, df = 189.4, p = 2.2e−16). Up to smoltification, fish in the Hatchery experiment grew much faster than fish in the River experiment. As expected, domesticated fish outgrew hybrids which outgrew wild fish, in both experiments [Table 1, strain significantly explained differences in weight in the Hatchery experiment (R^2^ = 0.7, p < 2.2e−16) and the River experiment (R^2^ = 0.42, p ≤ 2.2e−16)]. The Mowi fish produced in the River experiment (mean = 7371.7 g ± 786.7) were approximately twice as heavy as those produced in the Hatchery (mean = 3525.4 g ± 1536.5) (Table 1, t = − 17.38, df = 62.7, p = 2.2e−16) (note, this is primarily due to age differences).

**Table 1:** Number of spots (automatic counts within region of interest (ROI)) and weight (g) per strain

Origin	Experiment	Strain	n	No. of spots (n per ROI)			Weight (g)		
				Mean	Median	SD	Mean	Median	SD
Domesticated	Hatchery	Mowi	139	89.6	85.0	55.9	3525.0	3530.0	786.7
	River	Mowi	53	24.7	22.0	22.5	7372.00	7300.0	1536.5
Hybrid	Hatchery	Figgjo × Mowi	138	82.5	80.0	45.3	2239.0	2202.0	709.7
	Hatchery	Mowi × Figgjo	120	81.5	82.0	44.9	1922.0	1882.0	655.4
	River	Mowi × Etne	20	25.9	18.00	20.8	5755.0	5300.0	1722.1
Wild	Hatchery	Arna	111	49.3	48.0	30.7	889.0	870.0	379.5
	Hatchery	Figgjo	109	52.59	51.0	30.1	922.8	895.0	424.5
	Hatchery	Vosso	128	66.17	69.0	32.1	1192.0	1215.0	539.6
	River	Etne	112	31.7	28.0	15.4	4510.0	4350.0	1398.8

In both experiments, the number of spots correlated with fish size (Additional file 9: Figure A9.1) [R^2^ = 0.19, p < 2.2e−16 (Hatchery) and R^2^ = 0.07, p = 0.005 (River)]. Furthermore, fish weight and rectangular window size used to count the number of spots were strongly correlated (Hatchery, R^2^ = 0.94 p ≤ 2.2e−16, River, R^2^ = 0.94 p < 2.2e−16, Additional file 9: Figure A9.2). Therefore, to exclude the potentially biasing influence of fish size on estimates of spottiness, we adjusted the head spot counts for fish size using weight, and we adjusted the automatic spot counts using window size (ROI area), resulting in measures of spot density.

**Fig. 5:**
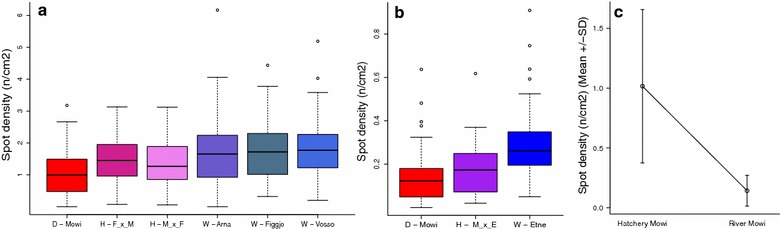
Spot density by strain. Fish from the Hatchery have a > 6.5-fold greater spot density than fish from the River experiment. Wild fish have greater spot density than domesticated fish. **a** The hatchery experiment by strain (n = 739) D = Domesticated (red), H = Hybrid (purple), W = Wild (blue), **b** the River experiment by strain (n = 182) D = Domesticated (red), H = Hybrid (purple), W = Wild (blue), **c** Mowi in the Hatchery (n = 139) vs Mowi in the River experiment (n = 53) (domesticated)

Wild fish displayed a greater spot density than hybrid and domesticated fish, in both experiments (Fig. 5a, b). Strain significantly explained spot density in the Hatchery experiment (R^2^ = 0.09, p = 6.7e−16) and the River experiment (R^2^ = 0.17, p = 2.7e−08). In the Hatchery experiment, hybrids were on average 1.4-fold spottier than domesticated salmon, and wild salmon were on average 1.7-fold spottier. In the River experiment, hybrids were 1.2-fold spottier and wild fish 1.8-fold spottier than the domesticated salmon. Nevertheless, differences in spot density among strains within environments were much smaller than the differences observed in spot density between the experiments. Regardless of genetic background, on average, fish from the Hatchery experiment (mean = 1.5 spots/cm^2^ ± 0.83) were 6.5-fold spottier than the fish from the River experiment (mean = 0.23 spots/cm^2^ ± 0.15) (Fig. 5a, b, significance t = 39.2, df = 880.4, p = 2.2e−16). Looking specifically at the domesticated strain, which was reared in both experiments, norm of reaction was used to illustrate the difference between the environments (Fig. 5c), The difference in spot density between the Hatchery experiment (mean = 1.02 spots/cm^2^ ± 0.64) and the River experiment (mean = 0.14 spots/cm^2^ ± 0.13) was sevenfold (significance: t = 15.28, df = 163.9, p = 2.2e−16). While the wild strains were all of different origin, the average fold difference in spot density between the Hatchery and River experiments was 6.2.

### Genetic basis of spot density

**Fig. 6:**
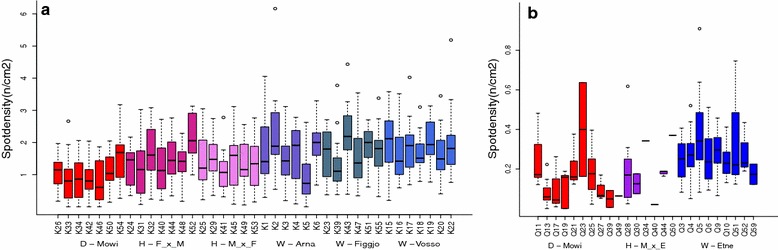
Spot density by family. **a** The Hatchery experiment (n = 739) D = Domesticated (red), H = Hybrid (purple), W = Wild (blue), **b** the River experiment (n = 182) D = Domesticated (red), H = Hybrid (purple), W = Wild (blue)

**Fig. 7:**
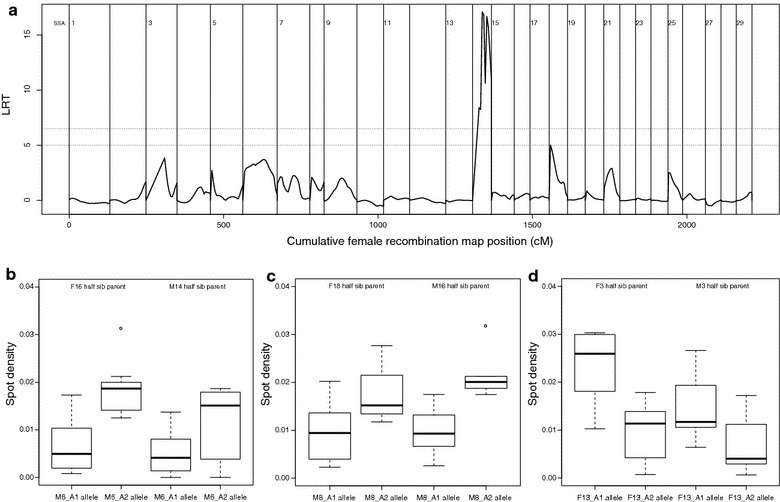
Results of QTL analysis. **a** Genome scan for QTL on spot density. The Likelihood Ratio Test (LRT) for QTL is plotted against all genomic positions tested (linkage groups are separated by vertical lines). The average spot density of the offspring is given for the offspring inheriting either allele from the parents contributing the QTL: **b** Mowi 6, **c** Mowi 8 and **d** Figgjo 13. As each parent was crossed with a domesticated (Mowi) and a wild (Figgjo) mate in a half sib family design, the offspring spot density is given in each background (farm/wild) separately. Figgjo 13 was determined by genotyping to be a farm escapee of unknown strain

We observed considerable variation in spot density among families within each strain (Fig. 6a, b). Fish strain and families were both significantly associated with variation in spot density: p < 2.21e−16 and p = 3.2e−6 respectively for strain and family in the Hatchery experiment, and p = 2.10e−4 and p = 0.014 respectively for strain and family in the River experiment. In a mixed model framework the spot density variance was split into two components: Strain and family, which were respectively associated with 11 and 7.3% of the total variance in the hatchery experiment and 10.9 and 8% in the River experiment. Heritability analysis within the Hatchery experiment computed a low narrow-sense heritability for number of spots (14%), spot density (6%), but a high heritability for sampling weight (54%). QTL analysis gave a significant (p < 0.01) hit on linkage group SSA014 for spot density (Fig. 7a). From the variance component analysis, the QTL in SSA014 explained 11% of the genetic variance, but only 1.3% of the total variance. The QTL was primarily caused by three individual parents, M6, M8, and F13 where inheritance of different alleles was clearly connected to significant differences in spot density in the progeny (Fig. 7b–d), (M6 t = 4.52, res. df = 36, p = 6.7e−5, M8 t = 6.60, res. df = 35, p = 1.1e−7, F13 t = 3.91, res. df = 30, p = 0.0004). KEGG analysis of the 4000 transcripts in the linkage group showed that five genes known to participate in melanogenesis (KO: 04916) are located here (ADCY1, ADCY2, ADCY8, EDNRB, MAP2K2), corresponding to 10 different sequences in the salmon genome predicted to code for these proteins (Additional file 8).

### Head spot counts and spots below the lateral line

Fish from the Hatchery experiment displayed an average of 10–15 head spots (13.3 ± 6.9), while fish from the River experiment displayed an average of six head spots (6.0 ± 3.1) (t test Hatchery expt. Vs. River expt. 2, t = 19.1, df = 389.5, p < 2.2e−16) (Additional file 9: Figure A9.3). Adjusted for fish weight, the trends per strain in the data also resemble those for spot density of the whole fish (Additional file 9: Figure A9.4 compared to Fig. 5). Head spot density and overall spot density were correlated in both datasets [Additional file 9: Figure A9.5, (a) Hatchery, R^2^ = 0.39 p < 2.2e−16. (b) River, R^2^ = 0.31, p = 3.6e−14]. Counts of spots below the lateral line were highly correlated with total spottiness in both experiments (Hatchery, R^2^ = 0.86 p < 2.2e−16. River, R^2^ = 0.92 p < 2.2e−16), Additional file 9: Figures A9.6, A9.7).

### Spot clustering

**Fig. 8:**
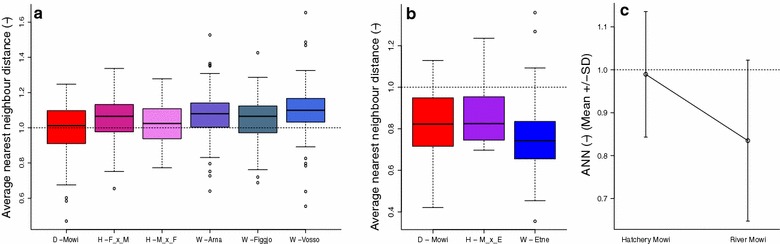
Clustering (ANN) by strain. Spot patterns are scattered in the Hatchery experiment and clustered in the River experiment. **a** The Hatchery experiment (n = 708) Domesticated = Mowi (red), Hybrid = Mowi × Figgjo (M × F), Figgjo × Mowi (F × M), (purple), Wild = Arna, Figgjo, Vosso (blue). **b** The River experiment (n = 161) Domesticated = Mowi (red), Hybrid = Mowi × Etne (M × E) (purple), Wild = Etne (blue). **c** Mowi in the Hatchery experiment (n = 130) vs Mowi in the River experiment (n = 38) (domesticated)

We calculated spot clustering using the average nearest neighbour distance (ANN). In both experiments, ANN was significantly correlated with strain but explained a very small part of the ANN variation (Fig. 8a), Hatchery, R^2^ = 0.02, p = 4.0e−4. (b) River, R^2^ = 0.08, p = 0.002. In contrast to the small differences in ANN observed among strains within environments, the average ANN in fish from the Hatchery experiment was > 1 (= scattered spots) (1.05 ± 0.14), while the mean ANN in fish from the River experiment was < 1 (clustered spots) (0.78 ± 0.18) (Fig. 8a, b). The difference in ANN between the two experiments was statistically significant (t = 16.8, df = 200.3, p = 4.5e−16), indicating that the environment caused not only differences in numbers and densities of spots (as above), but also in their clustering. The Mowi strain in the Hatchery experiment displayed random patterns (0.99 ± 0.15), while in the River experiment they displayed clustered patterns (0.83 ± 0.19) (t = 4.65, df = 50.88, p = 2.15e−5) (Fig. 8c).

## Discussion

This study provides the first experimental insight into the primary determinant of spotting patterns in one of the world’s most extensively studied fishes, the Atlantic salmon. We observed heritable variation and a significant QTL for spot density, demonstrating an underlying genetic influence for this trait. However, environmental variation was clearly the primary determinant as fish of domesticated, wild-domesticated F1 hybrid and wild pedigree all displayed a six to sevenfold higher density of spots when reared under farming conditions (Hatchery experiment—fish tanks, then sea cages), as opposed to a combination of the river and thereafter farming conditions (River experiment—natural river, then fish tanks). Different wild populations were used in the two experiments, potentially confounding the comparisons between environments. However, the domesticated Mowi strain was used in both experiments and showed a sevenfold higher density of spots in the Hatchery vs. the River experiment. We therefore conclude that environmental variation, as opposed to genetic variation, is the primary determinant of spot patterns in Atlantic salmon.

Our results beg two questions: what are the causative environmental triggers, and when are the fish receptive to these triggers? The available evidence from our study indicates that the diversity in spot patterns observed here were primarily set in the freshwater stage. Evidence supporting this includes: (1) Despite being reared in tanks for the marine phase, the fish reared in the natural river system up to smoltification (i.e. the River experiment) retained a clustered spot pattern with low spot density like that typically observed in salmon spending their entire lives in the wild [23, 39], (2) Kause et al. [40], reported no influence of freshwater vs saltwater-rearing on spot patterns in rainbow trout (*Oncorhyncus mykiss*) that were produced under identical rearing conditions until smoltification, (3) data from salmonids generally demonstrate that spot patterns are largely persistent over time, albeit with modifications [41–43]. Nevertheless, evidence suggesting that spot patterns can change during the marine phase can be obtained from a study of morphological differences between fish from farms and the wild [23]. These authors reported that salmon originating from six different farms displayed a greater number of spots than salmon originating from several rivers, a result consistent with ours. In contrast to the present study, however, these authors reported that salmon reared in tanks up to and including smoltification, then released into the sea to complete their marine phase of the life cycle in the wild, displayed spot patterns similar to wild salmon. In the present study, the fish reared under farming conditions were kept in sea cages post smoltification while the fish initially starting their life’s in the natural river system were held in tanks on land during the marine stage. This means that the exact environmental trigger that leads to spot pattern development cannot be determined from present evidence. Nevertheless, our data unequivocally demonstrate, for the first time, that environmental, as opposed to genetic variation, is the primary trigger for spotting patterns in this globally significant species.

Any combination of the environmental differences between freshwater rearing in the river and then marine rearing in tanks, vs. freshwater rearing in tanks and then marine rearing in open sea cages, may have triggered the diversity in spot patterns observed here. These could include, among other factors, light, diet, predation (selection), temperature, habitat, fish density and growth rate. Light is known to influence spot patterns in several ways [2]. 24-hour artificial lighting (typically lacking UV), is standard in salmon farming (including our study), in contrast to the situation in rivers. However, different artificial light regimes are not known to cause differences in spot patterns. Diet can affect melanisation [2]. Different farms and rivers have different dietary conditions, and current evidence suggests that the patterns form predictably regardless of this [23]. Salmon with many spots have been found to be less stress responsive and more dominant [11], and it is possible that this plays a role in the differences in pattern formation between the farm and the wild. However, a 14-week stress experiment in a hatchery did not result in pattern differences noticeable by eye [21]. The controlled conditions of fish farming are very well suited for further studies aiming to identify the trigger signal in Atlantic salmon.

With notable exceptions, current understanding of the genetics of spot pattern development in salmonids in general is sparse. Skaala and Jørstad demonstrated that the difference between fine and normally spotted brown trout is caused by a single unidentified gene displaying Mendelian inheritance [15]. Sivka et al. found that differences in spot patterns between brown trout and marble trout (*Salmo marmoratus*) is linked to differences in the expression of four genes involved in Wnt signalling, melanosome biogenesis and proopio-melanocortin (POMC) processing [44]. We found that narrow sense heritability was low for number of spots (14%) and spot density (6%), much lower than previously found for silver and spotty character in rainbow trout (23–45%) [13, 40]. Furthermore, we observed a significant QTL associated with spot density on linkage group SSA014. MC1R, a well-known determinant of degree of spotting in vertebrates [10], lies on linkage group SSA011 in Atlantic salmon. Thus, in our study, we did not observe a link between MC1R and the observed genetic variation. The linkage group covers over 4000 transcripts, but different isoforms of adenylate cyclase (ADCY1, ADCY2, ADCY8), endothelin B receptor (EDNRB) and MEK2 (MAP2K2) have predicted sequences within it, all of which are known participants in melanogenesis according to KEGG. Colouration has been shown to be regulated irrespective of both the sequence and expression level of MC1R in other poikilotherms [3]. Furthermore, in mammals, mutations in EDNRB causes a complete lack of spots, but in zebrafish this has been shown to only halve the number, which seems consistent with the effect seen here [3]. Thus, EDNRB is a good candidate for explaining our QTL hit.

Our results have an important application. Domestication of Atlantic salmon was first initiated in Norway in the early 1970s. As a result, domesticated salmon display genetic differences to wild salmon in a wide range of traits [19]. Each year, large numbers of domesticated Atlantic salmon escape from farms, and many find their way onto the spawning grounds of native populations. As a result, introgression of domesticated salmon has occurred in a large number of wild salmon populations [45, 46], and a reduction in population-genetic differentiation has been documented in Norway where this has been studied [47]. Therefore, identification, and subsequent removal of escapees from rivers is of importance to protect native populations from further impact. On the river bank, escapees are typically identified through deviating morphological characteristics such as fin erosion, spottiness and body condition [22, 23]. However, some of the morphological characteristics used for identification are plastic, and may partially or completely revert to the wild-type when they have been in the wild for an extended period of months or possibly years. Here, we provide further evidence that spot patterns, while primarily environmentally-induced, can be used to assist sorting farmed and wild fish in the rivers.

## Additional files

**Additional file 1.** Experimental design of crosses.

**Additional file 2.** Raw data.

**Additional file 3.** Methods—Image analysis macro.

**Additional file 4.** SNPs for QTL analysis.

**Additional file 5.** Phenotypes for QTL analysis.

**Additional file 6.** Genotypes for QTL analysis.

**Additional file 7.** Transcripts in QTL hit.

**Additional file 8.** Transcripts in KEGG hit.

**Additional file 9.** Data plots.

## References

[1] Maini PK (1997). Bones, feathers, teeth and coat markings: a unified model. Sci Prog.

[2] Leclercq E, Taylor JF, Migaud H (2010). Morphological skin colour changes in teleosts. Fish Fish.

[3] Mills MG, Patterson LB (2009). Not just black and white: pigment pattern development and evolution in vertebrates. Semin Cell Dev Biol.

[4] Sick S, Reinker S, Timmer J, Schlake T (2006). WNT and DKK determine hair follicle spacing through a reaction-diffusion mechanism. Science.

[5] Werner T, Koshikawa S, Williams TM, Carroll SB (2010). Generation of a novel wing colour pattern by the Wingless morphogen. Nature.

[6] Economou AD, Ohazama A, Porntaveetus T, Sharpe PT, Kondo S, Basson MA, Gritli-Linde A, Cobourne MT, Green JB (2012). Periodic stripe formation by a Turing mechanism operating at growth zones in the mammalian palate. Nat Genet.

[7] Kondo S, Miura T (2010). Reaction-diffusion model as a framework for understanding biological pattern formation. Science.

[8] Turing AM (1952). The chemical basis of morphogenesis. Proc Biol Sci.

[9] Bullara D, De Decker Y (2015). Pigment cell movement is not required for generation of Turing patterns in zebrafish skin. Nat Commun.

[10] Ducrest AL, Keller L, Roulin A (2008). Pleiotropy in the melanocortin system, coloration and behavioural syndromes. Trends Ecol Evol.

[11] Kittilsen S, Johansen IB, Braastad BO, Overli O (2012). Pigments, parasites and personality: towards a unifying role for steroid hormones?. PLoS ONE.

[12] Stringwell R, Lock A, Stutchbury CJ, Baggett E, Taylor J, Gough PJ, de Leaniz CG (2014). Maladaptation and phenotypic mismatch in hatchery-reared Atlantic salmon *Salmo salar* released in the wild. J Fish Biol.

[13] Colihueque N (2010). Genetics of salmonid skin pigmentation: clues and prospects for improving the external appearance of farmed salmonids. Rev Fish Biol Fisher.

[14] Skaala O, Jorstad KE (1987). Fine-spotted Brown Trout (*Salmo trutta*)—its phenotypic description and biochemical genetic-variation. Can J Fish Aquat Sci.

[15] Skaala O, Jorstad KE (1988). Inheritance of the fine-spotted pigmentation pattern of brown trout. Pol Arch Hydrobiol.

[16] Colihueque N, Araneda C (2014). Appearance traits in fish farming: progress from classical genetics to genomics, providing insight into current and potential genetic improvement. Front Genet.

[17] Garcia de Leaniz C, Fleming IA, Einum S, Verspoor E, Jordan WC, Consuegra S, Aubin-Horth N, Lajus D, Letcher BH, Youngson AF (2007). A critical review of adaptive genetic variation in Atlantic salmon: implications for conservation. Biol Rev.

[18] Gjedrem T (2010). The first family-based breeding program in aquaculture. Rev Aquac.

[19] Glover KA, Solberg MF, McGinnity P, Hindar K, Verspoor E, Coulson MW, Hansen MM, Araki H, Skaala O, Svasand T (2017). Half a century of genetic interaction between farmed and wild Atlantic salmon: status of knowledge and unanswered questions. Fish Fish.

[20] Glover KA, Ottera H, Olsen RE, Slinde E, Taranger GL, Skaala O (2009). A comparison of farmed, wild and hybrid Atlantic salmon (*Salmo salar* L.) reared under farming conditions. Aquaculture.

[21] Solberg MF, Skaala O, Nilsen F, Glover KA (2013). Does domestication cause changes in growth reaction norms? A study of farmed, wild and hybrid Atlantic salmon families exposed to environmental stress. PLoS ONE.

[22] Norwegian Veterinary Institute N. Salmopedia. In: Seafood Norway. 2014.

[23] Lund RA, Hansen LP, Järvi T. Identifisering av oppdrettslaks og vill-laks ved ytre morfologi, finnestørrelse og skjellkarakter. In: NINA Forskingsrapport 001. Ås: NINA; 1989. p. 1–54.

[24] Harvey AC, Glover KA, Taylor MI, Creer S, Carvalho GR (2016). A common garden design reveals population-specific variability in potential impacts of hybridization between populations of farmed and wild Atlantic salmon *Salmo salar* L. Evol Appl.

[25] Skaala O, Glover KA, Barlaup BT, Svasand T, Besnier F, Hansen MM, Borgstrom R (2012). Performance of farmed, hybrid, and wild Atlantic salmon (*Salmo salar*) families in a natural river environment. Can J Fish Aquat Sci.

[26] McGinnity P, Prodohl P, Ferguson K, Hynes R, O’Maoileidigh N, Baker N, Cotter D, O’Hea B, Cooke D, Rogan G (2003). Fitness reduction and potential extinction of wild populations of Atlantic salmon, *Salmo salar*, as a result of interactions with escaped farm salmon. Proc Biol Sci.

[27] Solberg MF, Fjelldal PG, Nilsen F, Glover KA (2014). Hatching time and alevin growth prior to the onset of exogenous feeding in farmed, wild and hybrid Norwegian Atlantic salmon. PLoS ONE.

[28] Skaala O, Glover KA, Barlaup BT, Borgstrom R (2014). Microsatellite DNA used for parentage identification of partly digested Atlantic salmon (*Salmo salar*) juveniles through non-destructive diet sampling in salmonids. Mar Biol Res.

[29] Solberg MF, Zhang Z, Nilsen F, Glover KA (2013). Growth reaction norms of domesticated, wild and hybrid Atlantic salmon families in response to differing social and physical environments. BMC Evol Biol.

[30] A language and environment for statistical computing. http://www.R-project.org/. Accessed 7 Feb 2018.

[31] Schneider CA, Rasband WS, Eliceiri KW (2012). NIH Image to ImageJ: 25 years of image analysis. Nat Methods.

[32] Mitchell A. The ESRI guide to GIS analysis, vol. 2. ESRI press; 2005.

[33] Besnier F, Glover KA, Lien S, Kent M, Hansen MM, Shen X, Skaala O (2015). Identification of quantitative genetic components of fitness variation in farmed, hybrid and native salmon in the wild. Heredity.

[34] Lien S, Gidskehaug L, Moen T, Hayes BJ, Berg PR, Davidson WS, Omholt SW, Kent MP (2011). A dense SNP-based linkage map for Atlantic salmon (*Salmo salar*) reveals extended chromosome homeologies and striking differences in sex-specific recombination patterns. BMC Genom.

[35] Pong-Wong R, George AW, Woolliams JA, Haley CS (2001). A simple and rapid method for calculating identity-by-descent matrices using multiple markers. Genet Sel Evol.

[36] Alam M, Ronnegard L, Shen X (2015). Fitting conditional and simultaneous autoregressive spatial models in hglm. R J.

[37] Churchill GA, Doerge RW (1994). Empirical threshold values for quantitative trait mapping. Genetics.

[38] Bates D, Machler M, Bolker BM, Walker SC (2015). Fitting linear mixed-effects models using lme4. J Stat Softw.

[39] Barlaup BT. Nå eller aldri for Vossolaksen. In: DN-Utredning 2008–9. vol. 9. Trondheim: Dir Nat; 2008: p. 172.

[40] Kause A, Ritola O, Paananen T (2004). Breeding for improved appearance of large rainbow trout in two production environments. Aquac Res.

[41] Merz JE, Skvorc P, Sogard SM, Watry C, Blankenship SM, Van Nieuwenhuyse EE (2012). Onset of melanophore patterns in the head region of Chinook Salmon: a natural marker for the reidentification of individual fish. N Am J Fish Manag.

[42] Gifford CM, Mayhood DW. Natural marks for identifying individual fish in small populations of at-risk Westslope cutthroat trout. In: Carline RF, LoSapio C, editors. Wild trout symposium IX: sustaining wild trout in a changing world; Bozeman, Montana. 2014. p. 275–81.

[43] Stien LH, Nilsson J, Bui S, Fosseidengen JE, Kristiansen TA, Øverli Ø, Folkedal O (2017). Consistent melanophore spot patterns allow long-term individual recognition of Atlantic salmon *Salmo salar*. J Fish Biol.

[44] Sivka U, Snoj A, Palandacic A, Susnik Bajec S (2013). Identification of candidate genes involved in marble color pattern formation in genus Salmo. Comp Biochem Physiol Part D Genom Proteom.

[45] Glover KA, Pertoldi C, Besnier F, Wennevik V, Kent M, Skaala O (2013). Atlantic salmon populations invaded by farmed escapees: quantifying genetic introgression with a Bayesian approach and SNPs. BMC Genet.

[46] Karlsson S, Diserud OH, Fiske P, Hindar K (2016). Widespread genetic introgression of escaped farmed Atlantic salmon in wild salmon populations. ICES J Mar Sci.

[47] Glover KA, Quintela M, Wennevik V, Besnier F, Sorvik AGE, Skaala O (2012). Three decades of farmed escapees in the wild: a spatio-temporal analysis of atlantic salmon population genetic structure throughout Norway. PLoS ONE.

