# Supplementary material for: Judging a salmon by its spots: environmental variation is the primary determinant of spot patterns in Salmo salar
Source: BMC Ecol. 2018 Apr 12;18:14. doi: 10.1186/s12898-018-0170-3 (PMC5897946; doi:10.1186/s12898-018-0170-3)
Supplement: Supplementary file 3 — Additional file 3. Methods—Image analysis macro. [file 12898_2018_170_MOESM3_ESM.pdf]

Additional file 3: ImageJ macro for image analysis.

ImageJ Macro

```
macro "SetScale [1]"{
run("Set Scale...", "known=228.1 unit=mm");
}

macro "MakeRectangle [2]"{
makeRectangle(1900, 1600, 1600, 400);
}

macro "Crop [3]" {
run("Crop");
}

macro "SaveAsTIF [4]" {
saveAs("Tiff");
}

macro "MeasureSpots [5]" {
run("8-bit");
//convert to greytone image
run("Remove Outliers...", "radius=50 threshold=50 which=Bright");
//remove bright reflexes
run("Auto Local Threshold", "method=Sauvola radius=100
parameter_1=0.7 parameter_2=128 white");
//local thresholding
run("Close-");
//connect particles that are very close
run("Options...", "iterations=1 count=1 do=Erode");
//remove very small particles (noise) and shrinks larger particles
run("Options...", "iterations=3 count=1 do=Dilate");
//Make particles grow
run("Watershed");
//Spots that are connected will be separated
run("Set Measurements...", "area centroid perimeter shape feret's
area_fraction display redirect=None decimal=2");
//Sets measurements types
run("Analyze Particles...", "size=2-Infinity show=Overlay display
exclude include add");
//Runs particle anlysis
run("Revert");
//Reverts thresholdes image to saved original
}
}

macro "RotateCW [6]"{
run("Rotate... ", "angle=2 grid=1 interpolation=Bilinear");
}

macro "RotateCCW [7]"{
run("Rotate... ", "angle=-2 grid=1 interpolation=Bilinear");
}
```

```
macro "Save ROI«s [8]" {
roiManager("Save","");

macro "Reset ROI«s [9]" {
roiManager("reset");
}
```
