# Supplementary material for: Judging a salmon by its spots: environmental variation is the primary determinant of spot patterns in Salmo salar
Source: BMC Ecol. 2018 Apr 12;18:14. doi: 10.1186/s12898-018-0170-3 (PMC5897946; doi:10.1186/s12898-018-0170-3)

# Additional file 9 Data plots

**Total spot count, automatic**

Total spot count is measured by ImageJ inside a defined area (region of interest (ROI)) centred about the lateral line and bounded by anatomical characteristics of each fish (see materials and methods). Fish with no spots are excluded from the analysis. Hatchery (n=739), River (n=182). Boxplots used are R default. Mowi = domesticated (red). FxM (Figgjo x Mowi), MxF (Mowi x Figgjo), MxE (Mowi x Etne) = hybrids (purple). Arna, Figgjo, Vosso, Etne = wild (blue).

Figure A.9.1 a) Total spot count versus ROI area correlated by strain: Hatchery

The number of spots can be expected to vary with fish size. Correlating total spot count with the area of the region of interest (ROI) used for measuring the spot count explores this.

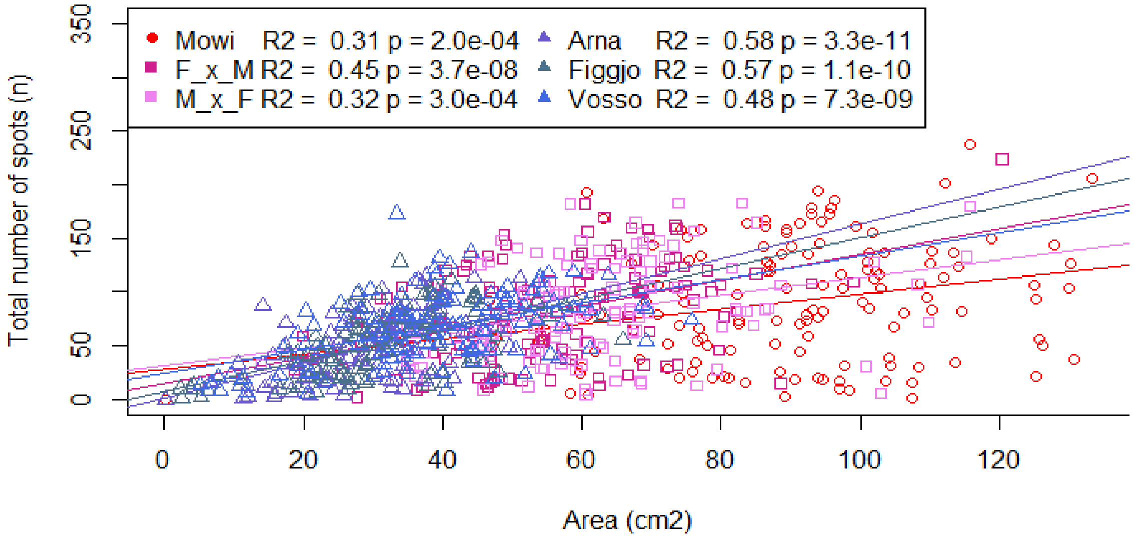


Figure A.9.1 b) Total spot count versus ROI area correlated by strain: River

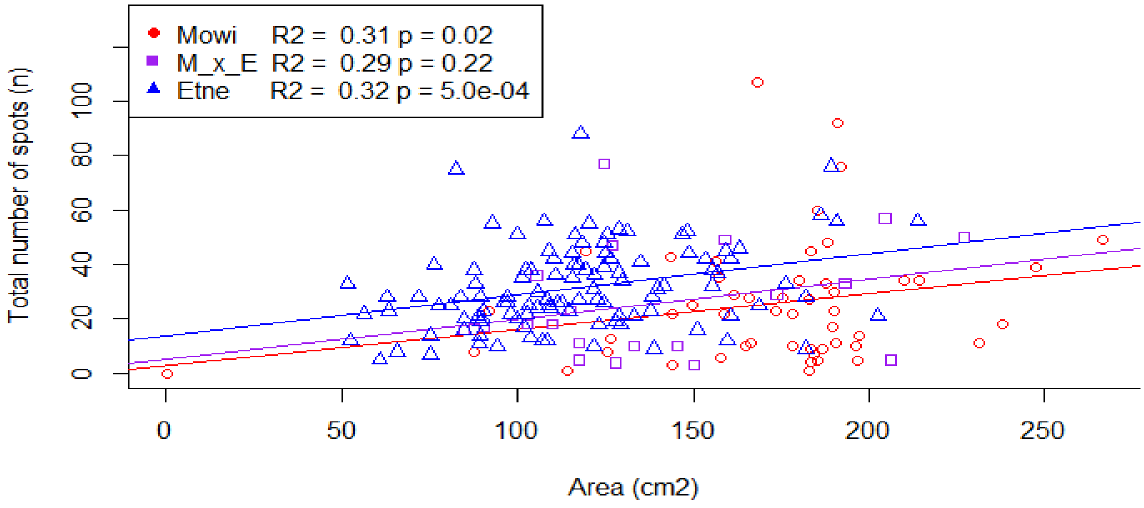


Total spot count correlates with fish size (ROI area) in all strains in both experiments (although in hybrids (MxE) in Expt. 2 this is not significant – which may be due to a low number of individuals (n = 20)). The relationship is strongest in wild Expt. 1 fish (largest $R^2$) (Arna,, Figgjo, Vosso)

**Weight**

Weight is the final sampling weight in both populations.
Hatchery (n = 745), River (n = 185).

Figure A9.2 Weight versus region of interest

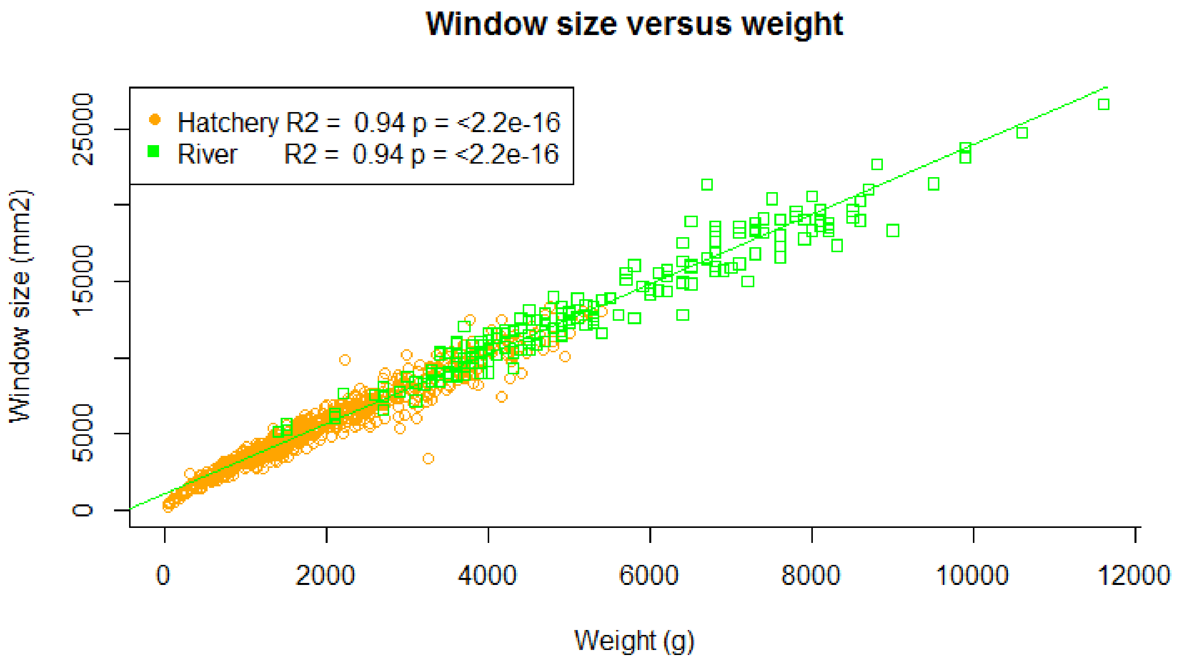


The fish are sampled for automatic spot counting and measuring using a region of interest (ROI) that is set relative to natural features in each fish. A strong correlation between ROI size and weight suggests that the sampled area is representative of fish size. Individuals with no spots were removed from this analysis as their ROI size cannot be measured, Hatchery (n=739), River (n=182) (fish with no spots excluded).

**Total head spot count**

Total head spot count is the sum of the gill to crease and crease to eye manual counts for each fish. Due to missing values (in a few images the whole or part of the head is outside the picture) the number of individuals are: Hatchery (n=741), River (n=131).

Figure A9.3 a) Total head spot count per strain: Hatchery

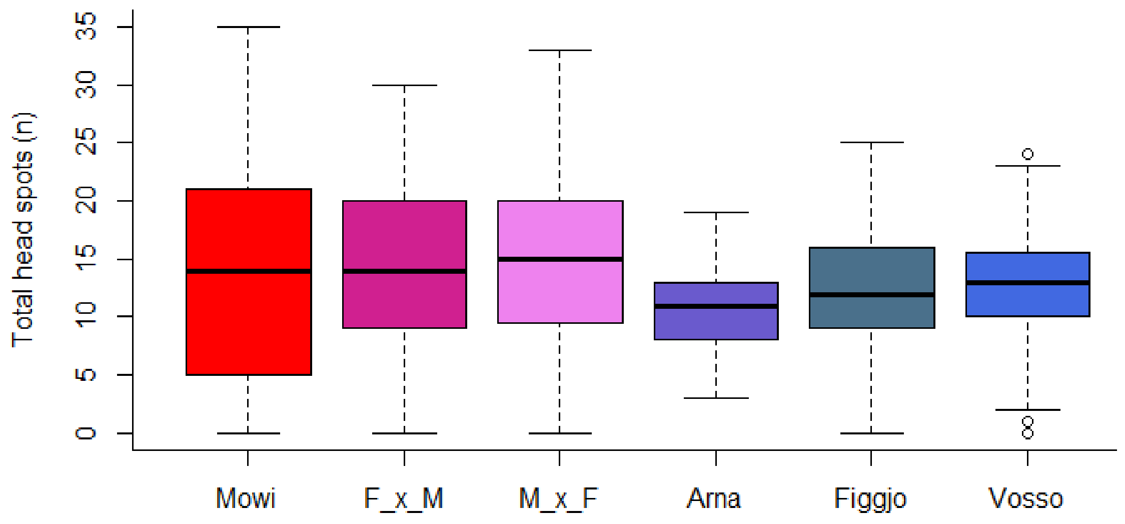


Figure A9.3 b) Total head spot count per strain: River, c) Hatchery vs River

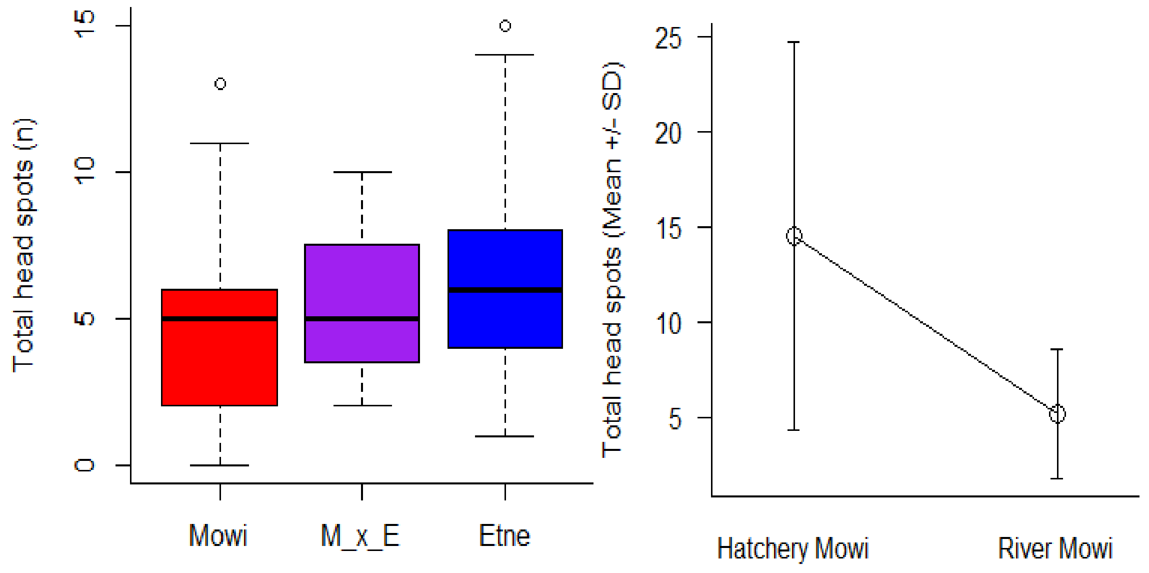


Despite the missing data especially in the crease to eye counts in the River dataset, the total head spot count gives an overall result that represents both results well – wild fish (Arna, Figgjo, Vosso, Etne) have more spots overall. The Hatchery result also resembles the individual area counts well. The difference between Hatchery Mowi (14.1 ± 10.3) vs River Mowi (5.0 ± 3.4) is significant ($t = 8.2$, $df = 116.01$, $p = 3.1e-13$).

Figure A9.4 a) Total head spot count per unit weight by strain: Hatchery

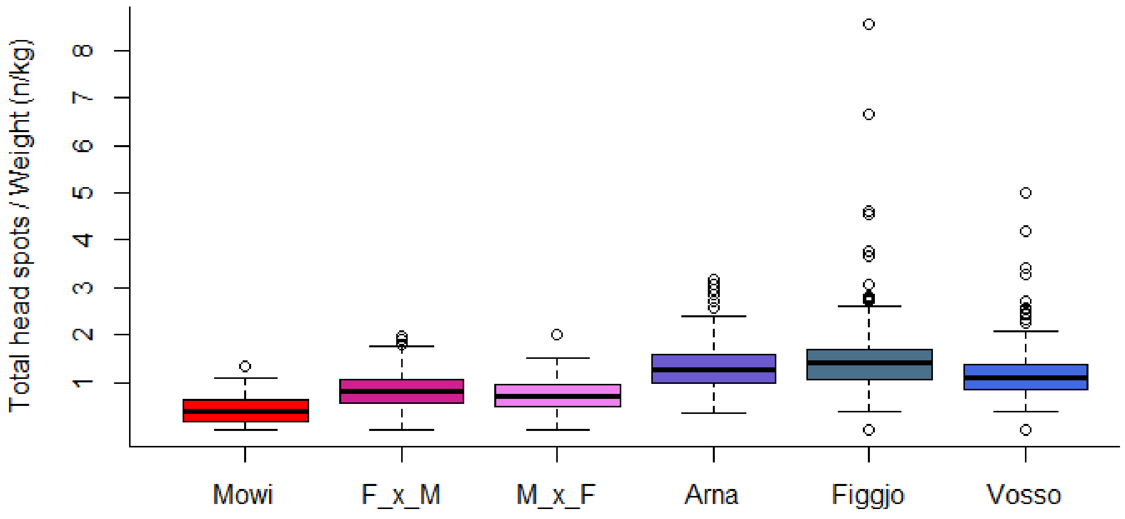


Figure A9.4 b) Total head spot count per unit weight by strain: River, c) Hatchery vs River

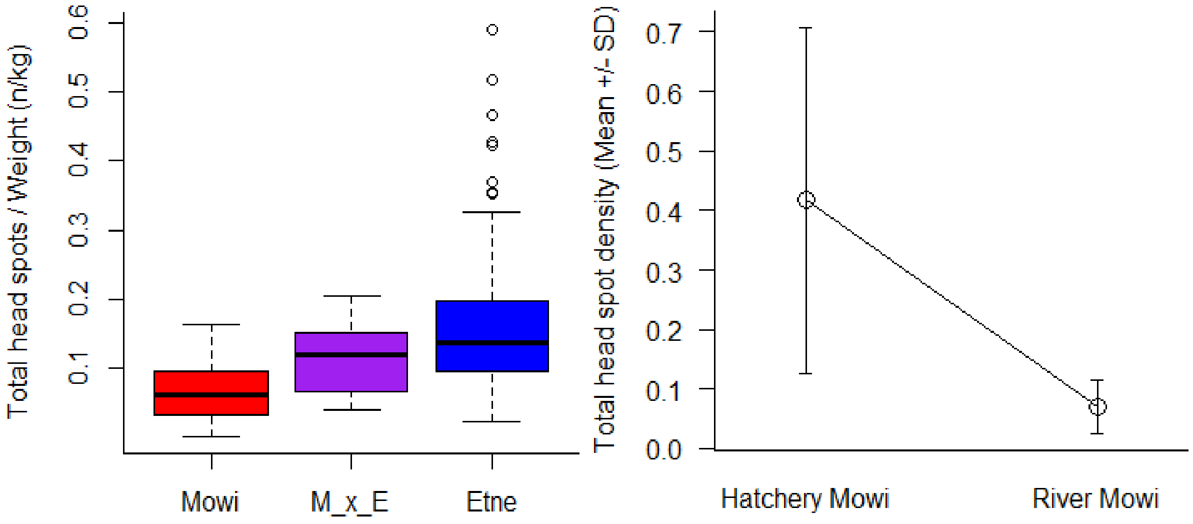


Wild fish (Arna, Figgjo,Vosso, Etne) have spottier heads compared to their body weight, and domesticated (Mowi) fish the least spotty, just like on their bodies. The difference between Hatchery Mowi (0.41 ± 0.3) vs River Mowi (0.07 ± 0.04) is significant (t = 12.7, df = 158.5, p = 2.2e-16).

Figure A9.5 a) Correlation of head spot density and overall spot density: Hatchery

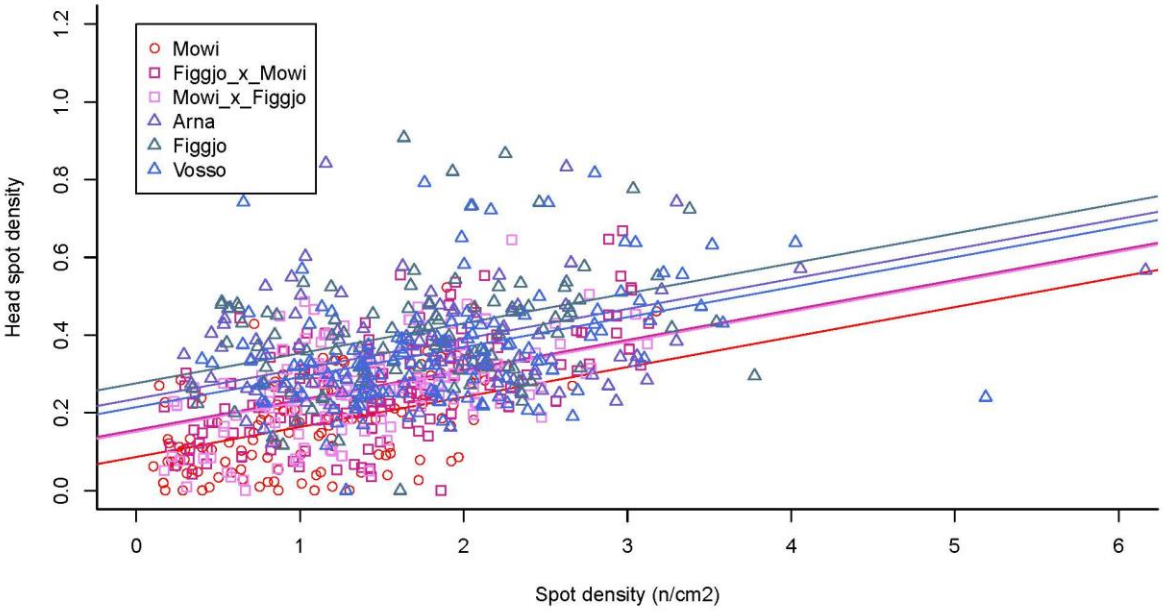


Figure A9.5 b) Correlation of head spot density and overall spot density: River

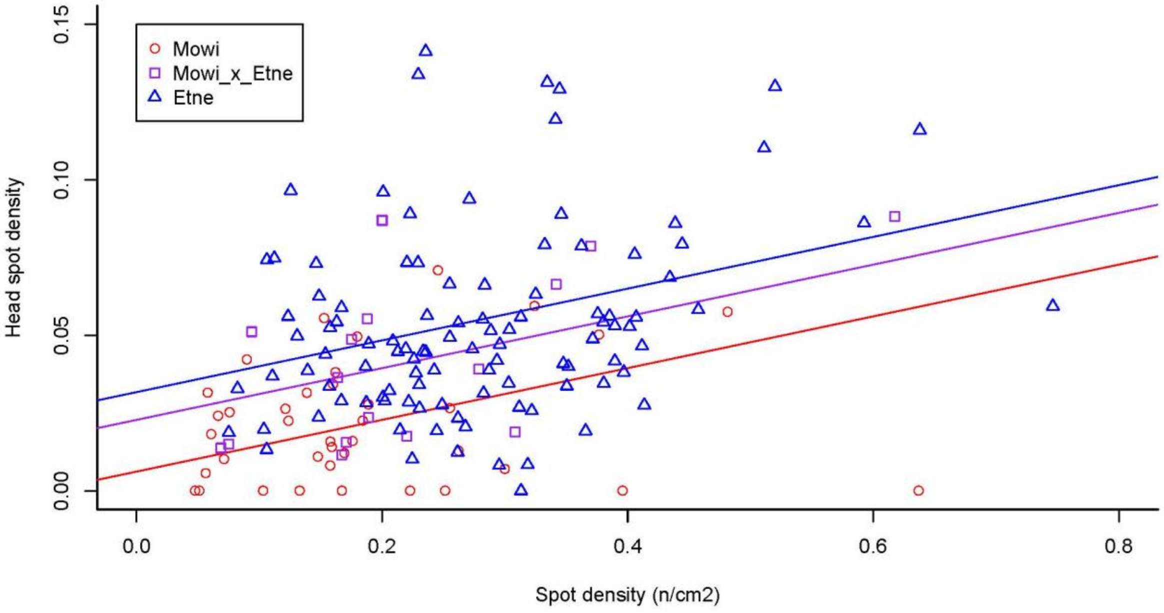


Strainwise correlations of head spot density and total spot density show a clear relationship, albeit with lower $R^2$ values in wild strains due mainly to greater variability in the number of head spots relative to total spots.

**Number of spots below lateral**

Number of spots below lateral is an automatic count within the selected region of interest (ROI). The ROI is centered about the lateral line of the fish, so this value is found by considering the spots in the lower half of the ROI. Fish with no spots were excluded from this analysis. Hatchery (n=739), River (n=182).

Figure A9.6 a) Spots below lateral (automatic) by strain: Hatchery

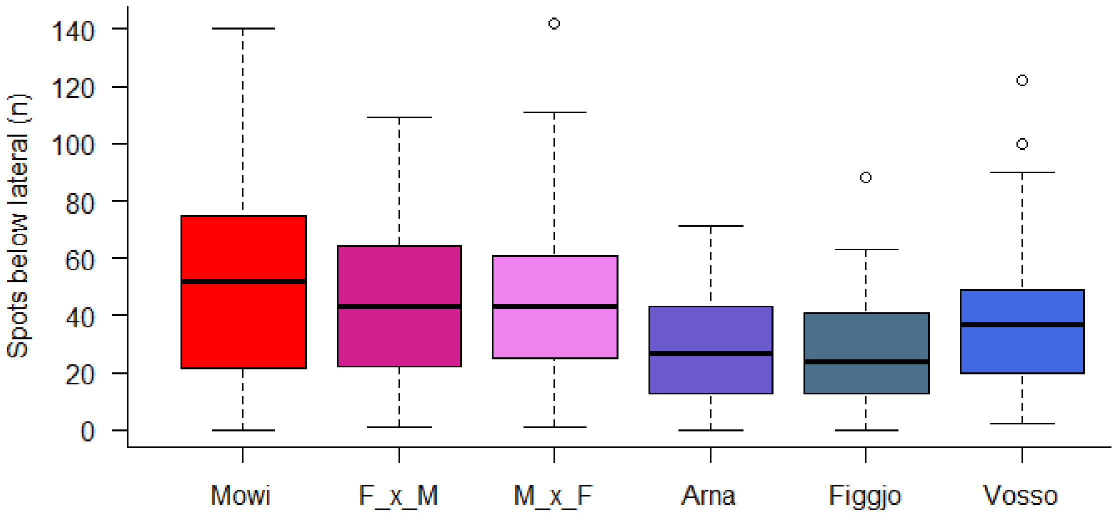


Figure A9.6 b) Spots below lateral (automatic) by strain: River c) Hatchery vs River

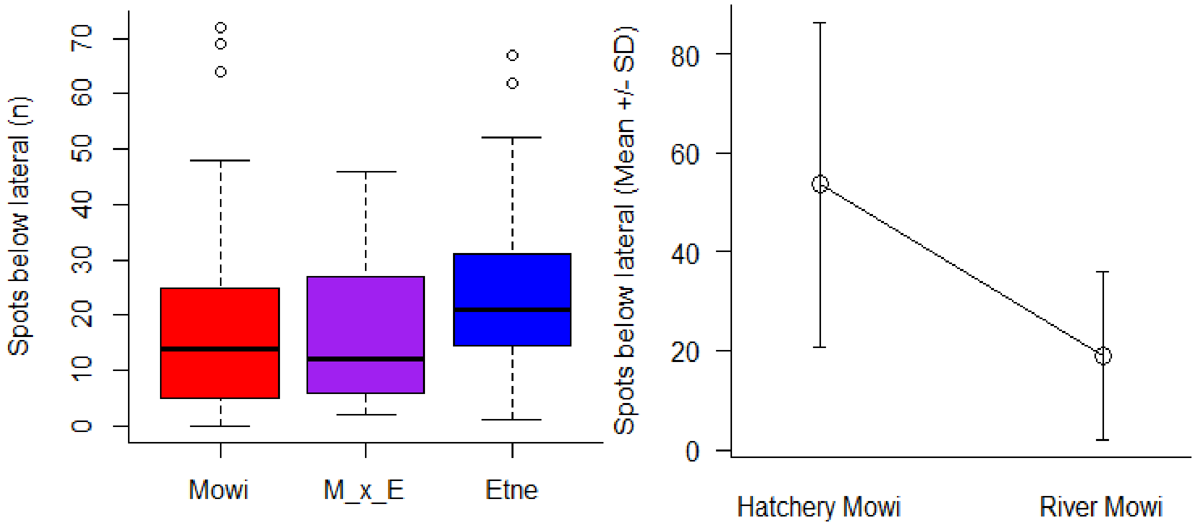


The number of spots below lateral is higher in domesticated (Mowi) and hybrid (MxF, FxM) fish in the Hatchery, while in the River it is the opposite – wild fish (Etne) have the highest number. This is very like the result for "number of spots" for the whole ROI area. The difference between Hatchery Mowi (53.4 ± 32.7) vs River Mowi (17.9 ± 17.01) is significant ($t = 9.69$, $df = 171.6$, $p = 2.2e-16$).

A9.7 a) Spots below lateral vs total number of spots: Hatchery

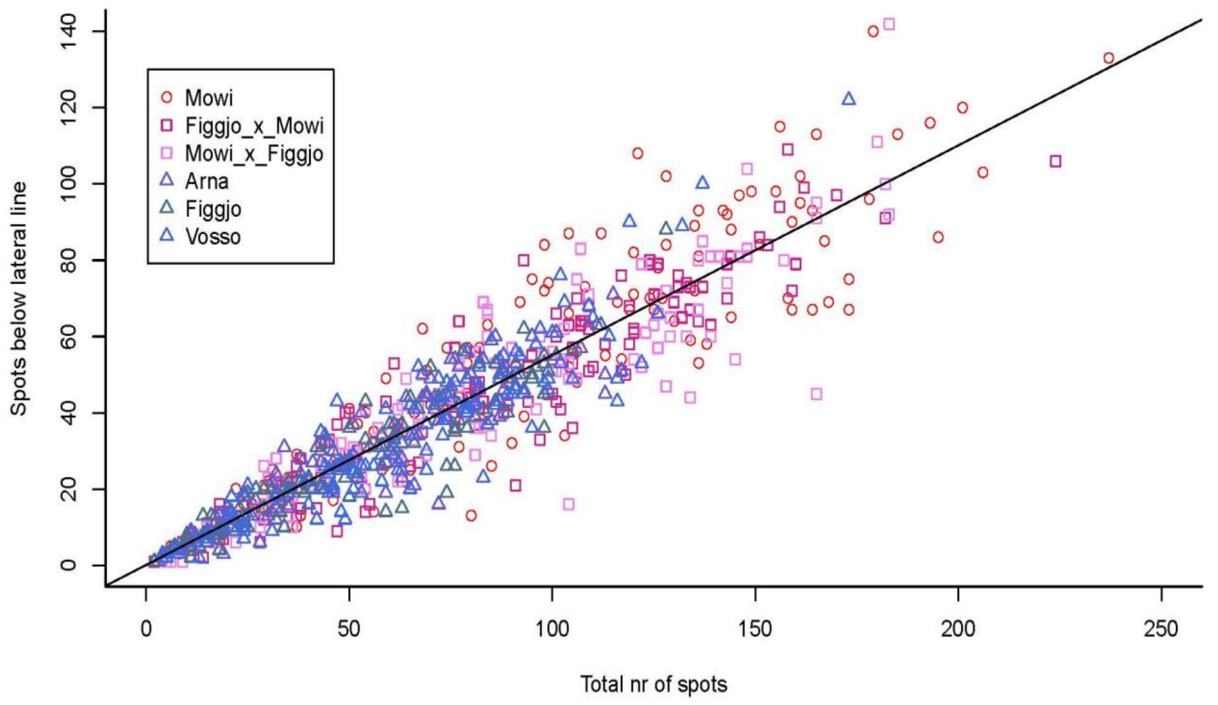


A9.7 b) Spots below lateral vs total number of spots: River

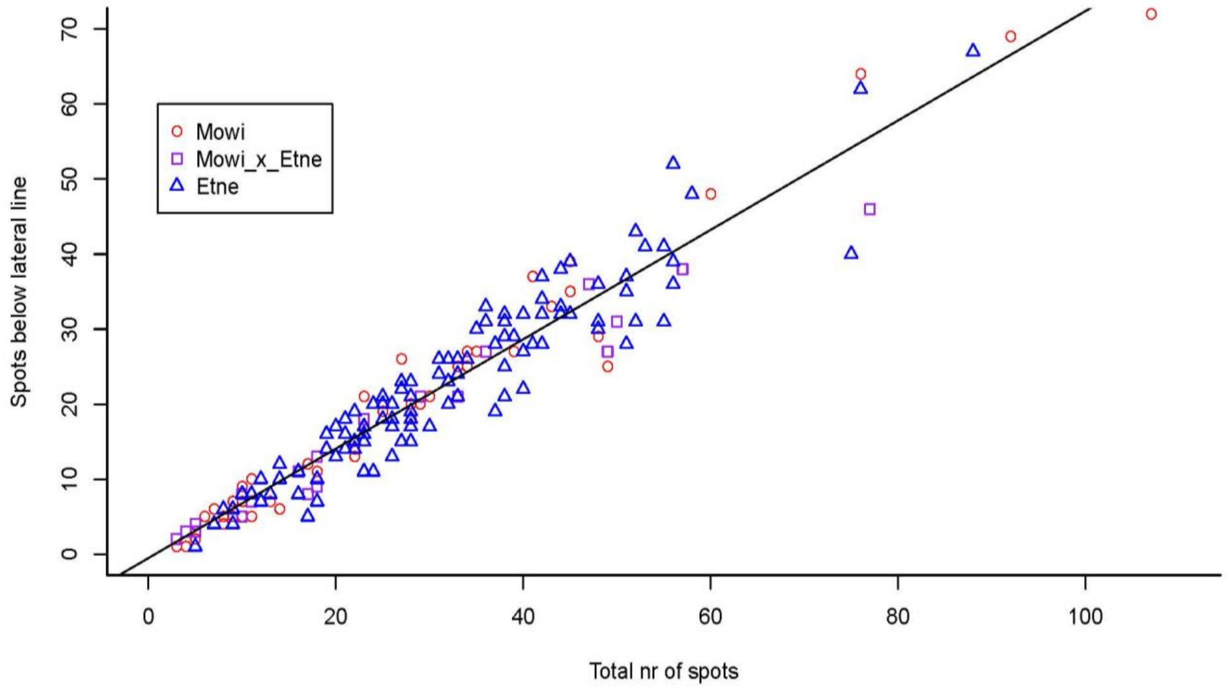

Supplement: Supplementary file 9 — Additional file 9. Data plots. [file 12898_2018_170_MOESM9_ESM.pdf]
